# A preliminary trial of botulinum toxin type A in patients with vestibular migraine: A longitudinal fMRI study

**DOI:** 10.3389/fneur.2022.955158

**Published:** 2022-07-25

**Authors:** Sun-Young Oh, Jin-Ju Kang, Sohui Kim, Jong-Min Lee, Ji-Soo Kim, Marianne Dieterich

**Affiliations:** ^1^Department of Neurology, Jeonbuk National University Hospital and School of Medicine, Jeonju-si, South Korea; ^2^Research Institute of Clinical Medicine of Jeonbuk National University-Biomedical Research Institute of Jeonbuk National University Hospital, Jeonju-si, South Korea; ^3^Department of Electronic Engineering, Hanyang University, Seoul, South Korea; ^4^Department of Biomedical Engineering, Hanyang University, Seoul, South Korea; ^5^Department of Neurology, Seoul National University College of Medicine, Seoul, South Korea; ^6^Department of Neurology, Dizziness Center, Clinical Neuroscience Center, Seoul National University Bundang Hospital, Seongnam-si, South Korea; ^7^Department of Neurology, University Hospital, Ludwig-Maximilians-Universität, Munich, Germany; ^8^German Center for Vertigo and Balance Disorders, University Hospital, Ludwig-Maximilians-Universität, Munich, Germany; ^9^Munich Cluster for Systems Neurology (SyNergy), Munich, Germany

**Keywords:** vestibular migraine, botulinum toxin (BOTOX^®^), prophylactic therapy, migraine-associated vertigo, migraine, vertigo, dizziness, headache

## Abstract

**Objective:**

This study aims to investigate the efficacy of botulinum toxin type A (BTX-A) in the prophylactic management of vestibular migraine (VM) and to determine whether this treatment modulates intrinsic functional brain network.

**Methods:**

Vestibular migraine patients (*n* = 20, mean age 45.4 years) who were resistant to conventional prophylactic therapies had BTX-A injection and rs-fMRI before and 2 months after the injection. We also measured the changes in the frequency of vertigo and migraine attacks, symptomatic functional disability scores, and neuropsychiatric inventories.

**Results:**

After BTX-A injection, the mean monthly frequencies of migraine and vertigo episodes decreased significantly compared with the baseline (*p* < 0.01, paired *t*-test). The Headache Impact Test-6 score and the Migraine Disability Assessment, and the vertigo parameters, measured by the Dizziness Handicap Inventory and the Vertigo Symptom Scale, showed an improvement, as did the anxiety and depression scores 2 months after BTX-A treatment. The low-frequency fluctuation analysis of the rs-fMRI data found significant changes in the functional connectivity of the right superior temporal gyrus. Adoption of this cluster as the seed region increased the functional connectivity with the left post-central gyrus, right supramarginal gyrus, and right middle temporal gyrus after BTX-A treatment.

**Conclusion:**

This prospective study suggests that BTX-A treatment is effective at ameliorating migraine and vertigo symptoms in VM patients who were resistant to conventional therapies. Along with symptomatic improvements, changes in the functional connectivity within the multisensory vestibular and pain networks suggest a dysmodulation of multimodal sensory integration and abnormal cortical processing of the vestibular and pain signals in VM patients.

## Introduction

The Bárány Society and the International Headache Society jointly proposed diagnostic consensus criteria for vestibular migraine (VM) included in the International Classification of Headache Disorders (ICHD-3) (Table 1) (1). VM is the most common cause of episodic vertigo in both adults and children, with a lifetime prevalence of 1% and a 1-year prevalence of 0.9% in the general population with a female preponderance (2). The underlying pathophysiology of VM is still unclear; however, possible mechanisms involve hyperactivity within the trigeminovascular system (TVS) and nociceptive brain stem centers, as well as an abnormal sensory modulation or integration in both pain and vestibular processing areas (3). Due to the absence of large, systematic, controlled clinical studies that specifically address VM, most therapeutic recommendations for VM are extrapolated from clinical trials on migraine with and without aura.

**Table 1:** Diagnostic criteria for vestibular migraine (1).

A. At least five episodes with vestibular symptoms of moderate or severe intensity ^a^ lasting 5 min to 72 h
B. Current or previous history of migraine with or without aura according to the ICHD-3
C. One or more migraine features with at least 50% vestibular episodes: a) headache with at least two of the following characteristics: - unilateral location - pulsating quality - moderate or severe intensity - aggravation by routine physical activity b) photophobia and phonophobia c) visual aura
D. Not better explained by another vestibular or ICHD diagnosis

a Vestibular symptoms are moderate when they interfere with but do not prevent daily activities and severe when they prevent such activities.

Botulinum toxin type A (BTX-A) has been used to treat different types of headache by blocking peripheral signals to the central nervous system, which inhibits central sensitization. The clinical efficacy of BTX-A in treating chronic migraine was previously demonstrated in the PREEMPT (Phase III Research Evaluating Migraine Prophylaxis Therapy) randomized clinical trial (4). BTX-A has also been used for other types of headaches, such as episodic migraine (<15 headache days per month) (5), chronic daily headache (>15 headache days per month) (6), and chronic tension-type headache, in a systematic series of exploratory controlled trials (7). In patients with frequent migraine headaches or vertigo attacks (15 days per month for more than 3 months), a trial of BTX-A might be reasonable; however, to date, there are no data on the management of chronic migraine headache or vertigo attacks in patients with VM.

Resting-state functional connectivity analysis in magnetic resonance imaging (rs-FC MRI) can visualize and measure the brain's intrinsic functional architecture, which aids in understanding the underlying mechanisms of various neurological disorders (8). The amplitude of low-frequency fluctuation (ALFF) changes in rs-FC MRI data is thought to be associated with local neuronal activity, and an ALFF analysis is effective for detecting fluctuations in spontaneous low-frequency oscillations.

The aim of this study was to evaluate the efficacy of BTX-A injection for the prophylactic treatment of VM. We hypothesized that a symptomatic improvement after BTX-A treatment would be reflected in changes in brain connectivity in areas that process and store vestibular and pain information. Therefore, rs-FC MRI data acquired pre- and post-BTX-A treatment were analyzed by comparing ALFF and seed-based rs-FC to detect changes in spontaneous brain activity in patients with VM.

## Subjects and methods

### Study design and participants

Patients at a dizziness clinic of Jeonbuk National University Hospital, Korea, who were diagnosed with VM using the criteria of the Headache Classification Committee of the International Headache Society (ICHD-3, appendix) and the Committee for the International Classification of Vestibular Disorders of the Bárány Society (9) were considered for inclusion in this study between May 2020 and January 2021 (Table 1). Twenty right-handed VM patients were included who suffered from moderate-to-severe vertigo attacks with migraine headache, with moderate impairment in daily life, despite treatment with several preventive drugs for at least 5 months. The use of migraine prophylactics was permitted if no changes in medications or dosages were made within the 2 months of study participation. Two or more categories of prophylactic medication were used prior to BTX-A injections in 75% (15/20) of patients. A calcium channel blocker (Nimodipine^®^) and topiramate were the two drugs most commonly prescribed in our patients; other preventive drugs used were cinnarizine, propranolol, valproate, and venlafaxine (antidepressant).

Patients were excluded if they had histories of medication overuse for headache or any medical condition or exposure to any agent that might have contraindicated the use of the BTX-A formulation, or if they had an infection or skin problem at any of the injection sites. Patients were also excluded if they had contraindications to MRI or had neurologic, psychiatric, audiovestibular, or systemic disorders or a history of complicated migraine attacks such as migrainous infarction or basilar migraine. Every patient underwent neurotological evaluations: video-oculography, video head impulse test, and cervical and ocular vestibular-evoked myogenic potentials. The structural and resting-state functional MRI scans were performed during interictal periods before (baseline) and 2 months after BTX-A injection therapy. Patients were treated with BTX-A (BOTOX^®^, Allergan, Inc., Irvine, CA, USA) injections based on the PREEMPT injection paradigm of fixed-site and fix-dose injections; a total of 155U was injected (5U each into 31 designated sites corresponding to seven muscles of the head and neck areas) (10).

This study was approved by the Ethics Committee of Jeonbuk National University Hospital (IRB File No. 2020-10-033-001), and all participants voluntarily signed written informed consent forms before entering the study.

### Efficacy measures

The primary efficacy measure was changes in the mean monthly frequency of vertigo and migraine headache episodes between the periods before and 2 months after the BTX-A injection. Headache or vertigo frequency was defined as calendar days on which a patient reported moderate to severe symptom episodes. The baseline for the efficacy measures was defined as the mean monthly frequency of vertigo or migraine episodes during the period of 2 months before the BTX-A injection.

We administered the following self-reported questionnaires before and after the treatment to gather data for our pre-specified secondary measure variables, which reflected mean monthly changes in the impact of headaches and vertigo on patient disability and psychiatric comorbidities: headache severity with visual analog scale (VAS), Headache Impact Test-6 (HIT-6), Migraine Disability Assessment (MIDAS) (11), Migraine-specific Quality of Life (MSQ), Vertigo Symptom Scale (VSS) (12), Dizziness Handicap Inventory (DHI) (13), Beck Depression Inventory (BDI), and Beck's Anxiety Inventory (BAI) (14).

### Imaging data acquisition and analysis

#### Structural and functional MRI

Structural and functional images were acquired on a 3T MRI system (Magnetom Verio, Siemens Healthcare, Erlangen, Germany) with a 12-channel head coil. In a single session, 195 volumes (60 contiguous, axial, 2.5-mm-thick slices; 1-mm gap) were acquired with a gradient echo, echo-planar imaging (EPI) T2^*^-sensitive sequence (repetition time: 2,000 ms; echo time: 30 ms; flip angle: 90°; matrix: 64 × 64; field of view: 192 × 192 mm). Anatomical images included a T1-weighted magnetization-prepared rapid gradient echo sequence with a 256-mm field of view and 1.0 × 1.0 × 1.0 mm3 isotropic spatial resolution (TE, 4.37 ms; TR, 2,100 ms; 160 slices). Resting-state fMRI data were preprocessed using SPM12 software (http://www.fil.ion.ucl.ac.uk/spm; Wellcome Trust Center for Neuroimaging, London, UK) implemented in Matlab^®^ 2020A (MathWorks^®^, Natick, MA). We performed slice timing correction using the standard SPM procedure, and for subsequent processing, we used the DPARSFA toolbox (data processing assistant for resting-state fMRI, version 5.1; https://www.nitrc.org/projects/dparsf) (15) together with the SPM software. A detailed methodology was described in the previous reports (16).

#### Amplitude of low-frequency fluctuations analysis

The ALFF analysis was performed using the DPARSF software. After the preprocessing described above, the fMRI data were temporally band-pass filtered (0.009 < *f* < 0.1 Hz) to reduce low-frequency drift and high-frequency respiratory and cardiac noise. The time series of each voxel was transformed into the frequency domain, and the power spectrum was obtained. Because the power of a given frequency is proportional to the square of the amplitude of that frequency component, the square root was calculated at each frequency of the power spectrum, and the average square root was then obtained across 0.009–0.1 Hz at each voxel. This average square root was taken as the ALFF, which was assumed to reflect the absolute intensity of spontaneous brain activity (17).

#### Defining regions of interest (ROIs) and the seed-based functional connectivity analysis

To investigate functional alterations in the VM patients, we performed a seed-based FC analysis. Significant pre- and post-treatment ALFF changes were demonstrated in the right superior temporal gyrus (STG) region; therefore, that right STG cluster was used as the seed for the FC analysis. The MNI coordinates of the center of the 5-mm spherical ROI were determined using the peak *t*-score detected at the right STG. For the FC analysis, the mean time series was extracted from the seed region and correlated with the time series of each voxel in the whole brain for each subject. The correlation coefficients were transformed into *z* values using Fisher's r-to-z transformation to improve normality. An entire brain *z*-score map was created for each subject.

### Statistical analyses

For each primary and secondary variable, the comparison between pre- and post-BTX-A treatment values was measured by an analysis of covariance in the change from baseline and the main effects of treatment. For all measures considered in this study, the data showed a normal distribution (Kolmogorov-Smirnov test, *p*>0.05). Paired *t*-tests were used to compare the baseline (pre-BTX-A treatment) values with the results obtained 2 months after the BTX-A treatment. The significance level was set to *p*<0.05, and the Statistical Package for Social Sciences software (SPSS, Inc., Chicago, IL, USA) was used for all data analyses.

To examine differences in the ALFF and FC between the baseline and follow-up scans, a paired *t*-test of the ALFF maps and z-score maps, respectively, was performed with age as a covariate. Statistical images were assessed for cluster-wise significance using a cluster-defining threshold of *p* < 0.05 with family-wise error (FWE) and false discovery rate (FDR) correction for multiple testing unless otherwise stated.

## Results

### Demographic and clinical characteristics

The demographic and clinical characteristics of the patient population are listed in Table 2. Twenty patients were enrolled; they were all women and right-handers with a mean age of 45 years (range from 20 to 69 years). The mean disease duration was 7.6 ± 4.8 years (range, 0.5–20 years), and the mean monthly frequency of migraine episodes at baseline was 17.2 ± 0.5, with a mean headache severity of 5.9 ± 1.3 (VAS). Patients had a mean frequency of 14.0 ± 5.0 episodes of vertigo per month. The vestibular symptoms lasted at least 1 h and had a mean duration of 11.3 h. Every patient experienced vestibular symptoms corresponding to a score of 4 or higher; the mean vertigo intensity was 6.0, and 7 patients had severe symptoms that interfered with their daily lives. Most subjects (16/20, 80%) had recurrent, spontaneous vertigo; they described a sensation of movement when no motion was taking place or an altered sensation without any provoking movements. In our patients' group, four patients complained of additional ear symptoms such as tinnitus (*n* = 3) and aural fullness (*n* = 1), which were not correlated temporally with symptoms of vertigo or headache. We believe that an overlapping syndrome of VM and Meniere disease in our case series was not included. Most participants revealed normal vestibular function tests, except one subject who revealed caloric weakness (39%) and decreased amplitude of cervical vestibular-evoked myogenic potentials on the right side, but other findings, including the head-impulse test and oculography, were unremarkable. Another patient showed subtle spontaneous nystagmus on interictal VOG recordings (about 3^°^/s) without caloric paresis or abnormal vHIT, which was resolved a month after the BTX injection.

**Table 2:** Demographic and clinical characteristics of patients with vestibular migraine (*n* = 20).

	**Patients (*n* = 20)**
Demographics	
Sex (female)	20 (100%)
Age (mean ± SD, range, years)	45.4 ± 12.8, 20–69
Right handedness	20 (100%)
Migraine characteristics	
Disease duration (mean ± SD, range, years)	7.6 ± 4.8, 0.5–20
Frequency (mean ± SD, range, days/month)	17.2 ± 9.5, 3–30
Severity of headache (VAS, 0–10) (mean ± SD, range)	6.8 ± 1.3, 4–8
Episode duration (mean ± SD, range, h)	17.3 ± 19.0, 1–72
MwoA / MwA / Visual aura	13 (65%) / 7 (35%) / 7 (35%)
Headache side: right / Left / Bilateral	1 (5%) / 6 (30%) / 13 (65%)
Pulsating quality	7 (35%)
Photophobia / Phonophobia	2 (10%) / 4 (20%)
Vestibular symptoms	
Frequency (mean ± SD, range, days/month)	14.0 ± 5.0, 2–21
Duration (mean ± SD, range, h)	11.35 ± 16.9, 1–72
Intensity of vertigo (VAS-D, 0–10) (mean ± SD, range)^a^	6.0 ± 2.8, 4–10
Recurrent spontaneous / positional / visually induced vertigo	16 (80%) / 9 (45%) / 3 (15%)
Nausea or vomiting	14 (70%)
History of motion sickness	12 (60%)
Frequency of acute headache/vertigo treatment use (days/month)	15.0 ± 10.7
Prophylactic medications	20 (100%)
Use of two or more drugs	15 (75%)
Vestibular function tests	
Spontaneous nystagmus (VOG)	1 (5%)
vHIT (hVOR mean gain, R/L)	0.97 ± 0.05 / 0.98 ± 0.06
Abnormal vHIT hVOR gain / Presence of corrective saccades	0 / 0
VEMP (cVEMP, oVEMP)	
cVEMP p13 amplitude AR ≥ 40%	1 (5%)
cVEMP p13 amplitude AR (%)	20.19 ± 12.40
cVEMP p13 mean latency (ms, R/L)	13.17 ± 0.4/12.91 ± 0.5
oVEMP n10 amplitude AR ≥ 40%	0
oVEMP n10 amplitude AR (%)	18.42 ± 11.70
oVEMP n10 mean latency (ms, R/L)	10.11 ± 0.7/10.39 ± 0.5
Mean caloric response (°/s, R/L)	28.6 ± 15.4/26.4 ± 12.3
Caloric response AR	15.61 ± 21.9
Caloric response AR ≥ 35%	1/7 (14.3%)

AR, asymmetry ratio; vHIT, video head-impulse test; VOG, video oculography; VOR, video ocular reflex; VAS, Visual Analog Scale; VAS-D, Visual Analog Scale-Dizziness; R, right; L, left.

a To evaluate the intensity of vestibular symptoms, we used a scale similar to the numeric rating scale, with 0 set as none, 1–3 as mild (uninterrupted), 4–6 as moderate (disturbed but able to maintain daily activities), and more than 7 as severe (daily activities cannot be continued).

### Efficacy measures after BTX-A treatment

There was an overall reduction in the mean monthly frequency of headache and vertigo episodes 2 months after BTX-A treatment compared with baseline (Table 3). During the prospective baseline phase, VM patients had a mean monthly frequency of 17 migraine headaches and 14 vertigo episodes, which were significantly reduced to mean monthly frequencies of 9.5 (−8.3 days, *p* = 0.001, paired *t*-test) and 8.5 (−5.5 days, *p* < 0.001), respectively, 2 months after BTX-A injection. Some patients, however, did not show the effect of BTX-A treatment as <30% reduction (*n* = 3), no change (*n* = 2), or increase (*n* = 2) in the headache days per month.

**Table 3:** Efficacy of botulinum toxin type A injection.

**Parameters**	**Baseline**	**After treatment**	***p*** **-value** ^**a**^
Headache profile			
Frequency (days/month)	17.8 ± 9.9	9.5 ± 10.1	0.001
Severity (VAS, 1–10)	6.8 ± 1.3	5.0 ± 2.6	<0.001
HIT-6 (6 questions, 78 points)	65.0 ± 7.4	55.2 ± 8.8	<0.001
Severe headache (HIT-6 ≥ 60 points)	17 (85%)	7 (35%)	0.002^b^
MIDAS (5 questions, days/3 month)	53.3 ± 29.4	28.3 ± 30.3	0.001
grade 1 (MIDAS score 0–5)	0 (0%)	8 (40%)	0.002^b^
grade 2 (MIDAS score 6–10)	0 (0%)	2 (10%)	
grade 3 (MIDAS score 11–20)	3 (15%)	0 (0%)	
grade 4 (MIDAS score ≥21)	17 (85%)	10 (50%)	
MSQ (14 questions, 100 points)	68.8 ± 47.1	105.0 ± 40.3	<0.001
MSQ_RR (7 questions)	23.9 ± 15.0	36.1 ± 13.7	0.001
MSQ_RP (4 questions)	24.3 ± 15.8	34.5 ± 14.4	0.003
MSQ_EF (3 questions)	20.7 ± 18.1	34.3 ± 16.8	0.001
Vestibular symptom profiles			
Frequency (days/month)	14.0 ± 5.0	8.5 ± 5.4	<0.001
Intensity (VAS-D, 0–10)	6.0 ± 2.8	3.7 ± 2.7	0.001
VSS (15 questions, 60 points)	30.1 ± 10.5	18.2 ± 11.3	<0.001
DHI (25 questions, 100 points)	52.4 ± 27.8	31.6 ± 22.9	<0.001
Physical (7 questions, 28 points)	11.6 ± 6.6	7.9 ± 5.2	0.001
Functional (9 questions, 36 points)	20.1 ± 11.9	13.8 ± 10.5	0.015
Emotional (9 questions, 36 points)	19.2 ± 10.0	10.1 ± 9.0	<0.001
Depression and anxiety profile			
BDI (21 questions, 63 points)	23.9 ± 15.6	16.0 ± 11.0	<0.001
BAI (21 questions, 63 points)	26.6 ± 14.7	16.7 ± 13.0	0.001

VAS, visual analog scale; HIT-6, Headache Impact Test-6; MIDAS, Migraine Disability Assessment; MSQ, Migraine Specific Quality of Life; RR, Role-function Restrictive; RP, Role-function Preventive; EF, Emotional Function; VAS-D, Visual Analog Scale-Dizziness; VSS, Vertigo Symptom Scale-short form; DHI, Dizziness Handicap Inventory; BDI, Beck Depression Inventory; BAI, Beck Anxiety Inventory.

a Paired t-test;

b Pearson's chi-square test.

**Figure 1:**
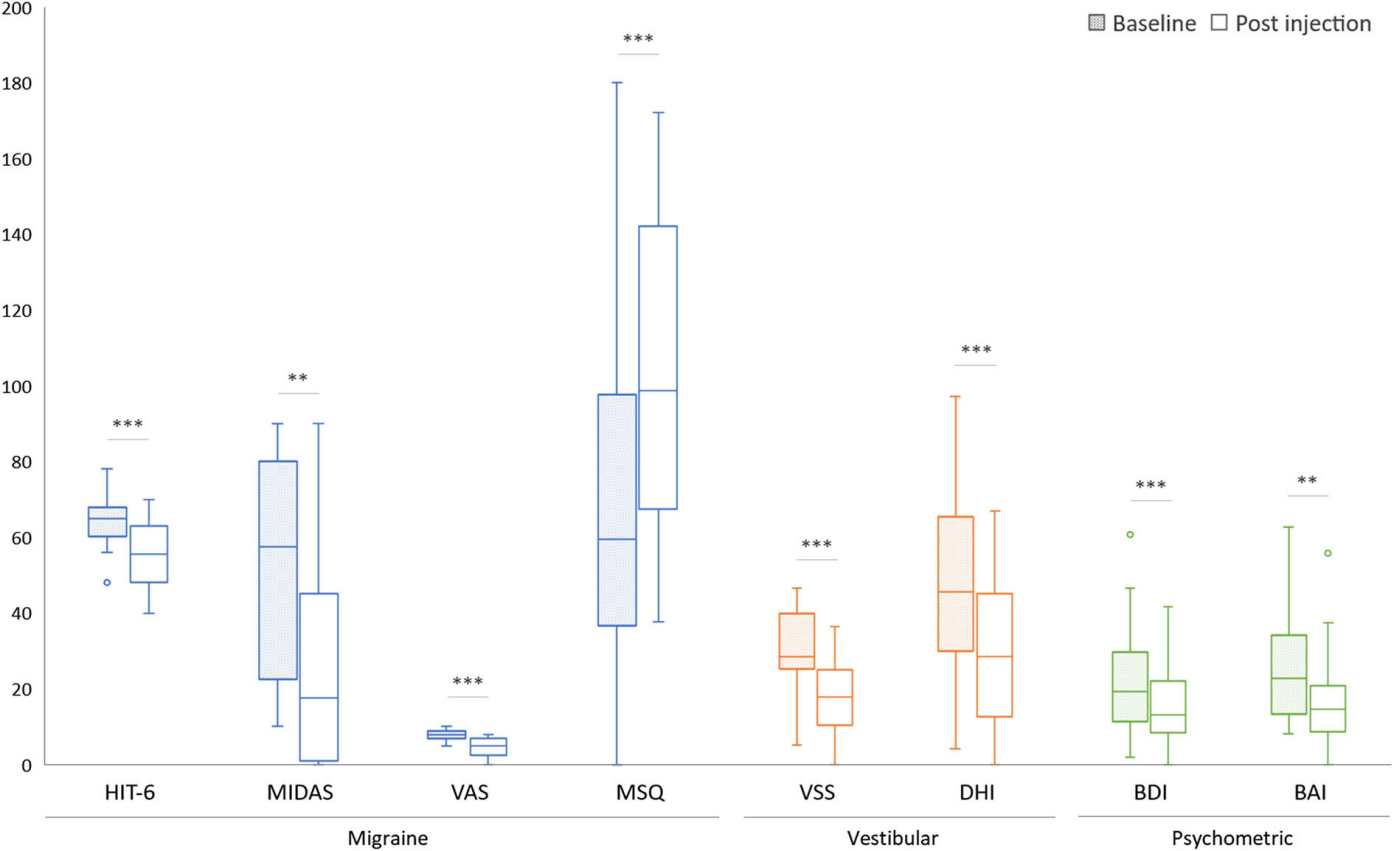
Comparison of symptomatic functional disability scores before (baseline) and after BTX-A treatment. ^*^p < 0.05, ^**^p < 0.01, ^***^p < 0.001.

Both the headache and vertigo profiles of our VM patients showed marked improvement after the BTX-A treatment (Table 3, Figure 1). The HIT−6 (−9.5, 95% CI, −13.1 to −5.9, *p* < 0.001, paired *t*–test), the MIDAS (−25.2, 95% CI, −38.0 to −14.2, *p* = 0.001), and the MSQ scores (+36.2, 95% CI, 20.6–53.2, *p*<0.001) were significantly improved after the BTX–A treatment. In the vertigo profiles, the VSS (−12.6, 95% CI, −18.1 to −8.0, *p* < 0.001, paired *t*–test) and the DHI (−20.1, 95% CI, −27.1 to −13.1, *p* < 0.001) were also improved after the injection. The mean scores of the BDI (−6.9, 95% CI, −10.3 to −3.5, *p* < 0.001) and BAI (−9.1, 95% CI, −14.0 to −4.5, *p* = 0.001) were improved 2 months after the BTX-A injection.

### Resting-state ALFF values after BTX-A treatment

**Figure 2:**
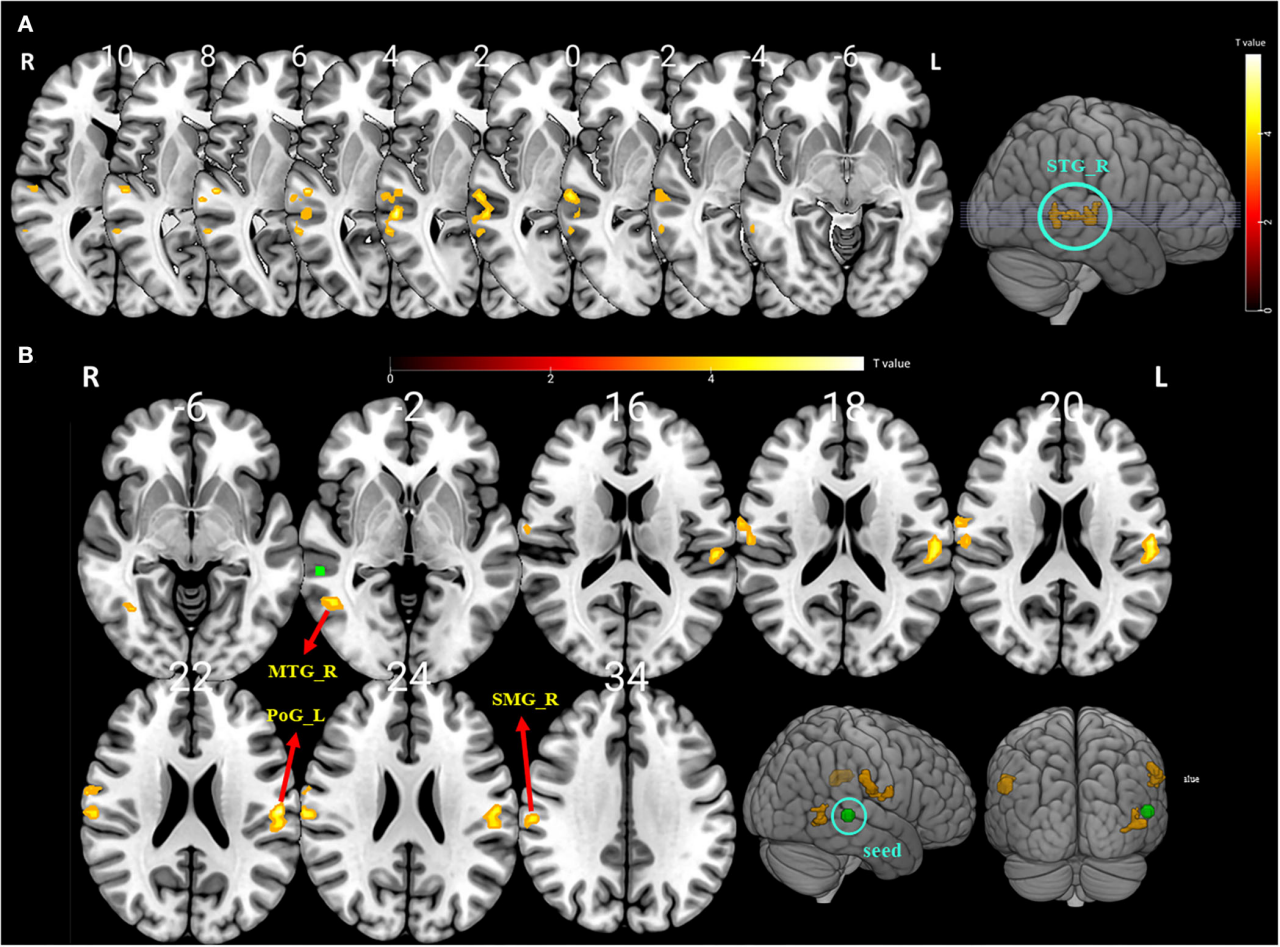
**(A)** The significantly altered amplitude of the low-frequency fluctuation (ALFF) map of the right superior temporal gyrus (STG) in vestibular migraine patients after BTX-A treatment (FDR and FWE *p* < 0.05 corrected). The color bar denotes the *t*-value. **(B)** Functional connectivity (FC) analysis using the peak of the overlapped cluster at the right STG (5-mm green sphere; 57, −36, 3) as the seed. Positive (hot color) *t*-value indicates increased FC (i) between the right STG and right supramarginal gyrus (SMG); (ii) between the right STG and left postcentral gyrus (PSG); and (iii) between the right STG and right middle temporal gyrus (MTG) (FDR and FWE *p* < 0.05 corrected).

Compared with the baseline, the ALFF values were significantly higher in the right STG after BTX-A treatment (*p* < 0.001, FDR and FWE corrected, Table 4 and Figure 2A).

**Table 4:** Brain regions with significant amplitude of low frequency fluctuation (ALFF) differences between pre- and post-BTX-A injection in vestibular migraine patients.

**Cluster**	**Brain region**	**Cluster size**	**x**	**y**	**z**	***t*** **value**
Cluster 1	Right superior temporal gyrus	67	57	−36	3	5.94
			54	−21	3	5.61
			63	−24	−3	5.39

ALFF for contrast post-treatment > pre-treatment (FDR and FWE p < 0.05 corrected).

### Seed-based functional connectivity

**Figure 3:**
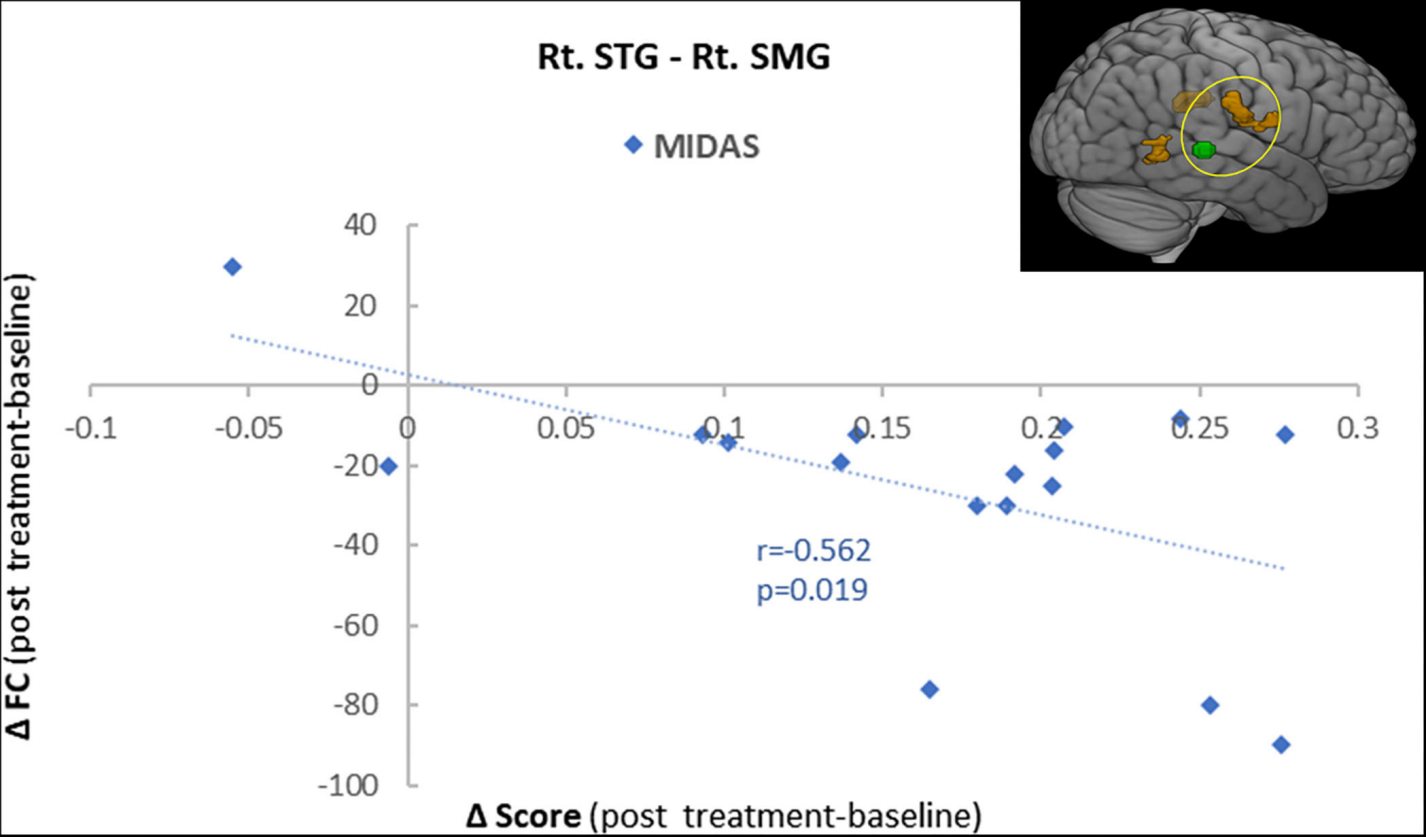
A correlation analysis showed a significant negative correlation between the functional connectivity of the right superior temporal gyrus (STG) with the supramarginal gyrus (SMG) and the MIDAS score (*r* = −0.56, *p* = 0.001).

To further explore the role of the right STG in VM management using BTX-A injection, we applied an FC analysis using the peak of the overlapped cluster at the right STG (57, −36, 3, 5 mm) as the seed. After BTX-A treatment, the following FC values were increased between the right STG and (i) right supramarginal gyrus (SMG); (ii) left postcentral gyrus (PCG); (iii) right middle temporal gyrus (MTG) (Figure 2B, Table 5). Subanalyses revealed a significant negative correlation between the FC of the right STG–SMG and the migraine disability MIDAS (*r* = −0.562, *p* = 0.019, Pearson's correlation analysis) (Figure 3), and no other relevant subanalysis showed significant correlations.

**Table 5:** Results from the seed-based functional connectivity analysis showing altered right superior temporal gyrus (STG) functional connectivity (FC) pre- and post-treatment across patients.

**Cluster**	**Brain region**	**Cluster size**	**x**	**y**	**z**	***t*** **value**
Cluster 1	Right supramarginal gyrus (SMG)	62	63	−24	30	5.9
			57	−21	36	4.96
			66	−6	21	4.87
Cluster 2	Left postcentral gyrus (PCG)	51	−57	−18	21	6.08
Cluster 3	Right middle temporal gyrus (MTG)	33	51	−57	−3	5.38
			36	−60	−6	4.72
			48	−57	6	4.67

FC with right STG for contrast post-treatment > pretreatment (FDR and FWE p < 0.05 corrected).

## Discussion

In this study, 155U BTX-A injections significantly reduced both vertigo attacks and migraine headache compared with baseline in our 20 patients with VM. The patients also reported significant improvements in their functionality and the tolerability of migraine headache and vestibular symptoms after BTX-A treatment.

Although various mechanisms have been put forward to explain VM symptoms, interactions between nociceptive and vestibular pathways are broadly considered to represent the underlying pathophysiology of VM. The reciprocal connections between the vestibular nuclei and other brainstem structures, such as the raphe nuclei, the parabrachial nucleus, and the locus coeruleus, could modulate the sensitivity of the trigeminovascular system (TVS) and pain pathways (18), which could explain vestibular symptoms in VM. Altered neural activity within the TVS is considered to be the primary mechanism for migraine headache (19). Thus, TVS neuropeptides such as substance P and calcitonin gene-related peptide (CGRP), which cause vasodilation and neurogenic inflammation, might lead to throbbing pain and central sensitization (20). The TVS also innervates the inner ear, and some neurotransmitters, such as CGRP and serotonin, are expressed in the vestibular and cochlear systems and might also be involved in VM pathophysiology (21). Animal studies have shown increased CGRP expression in a rotational model of motion sickness (22), and CGRP null mice have a reduced vestibulo-ocular reflex (23), which gives more evidence that CGRP is expressed throughout various vestibular nuclei (24). These findings highlight the potential role of elevated CGRP signaling as an underlying mechanism in the association between migraine and vertigo (25). Parallel activation of the vestibular and nociceptive pathways, such as the periaqueductal gray and hypothalamic areas, has been proposed, and reciprocal connections between these systems could modulate neural activity within the TVS and vestibular system (26, 27). Furthermore, the response of both headache and vertigo symptoms to migraine prophylactic medications suggests that migraine headache and vertigo could share a common pathophysiology in the VM population (26).

Although the exact mechanism of action of BTX-A in VM is still a matter of speculation, animal studies suggest that BTX-A can block the release of nociceptive mediators such as substance P, glutamate, and CGRP, which reduces nociceptive input into the central nervous system from the periphery (28). Correspondingly, it has been shown that CGRP levels in chronic migraine patients strongly correlate with their response to BTX-A therapy (29), and CGRP levels after BTX-A treatment were significantly lower than before BTX-A treatment (median, 51.89 vs. 74.09 pg/ml, *p*<0.001) in patients with chronic migraine (30). Therefore, the activation of the TVS and vestibular system and the release of pain-producing molecules, especially CGRP, could be a potential therapeutic intervention during chronic migraine and VM attacks. Injections of BTX-A at the designated pericranial sites inhibit the release of CGRP, which would otherwise facilitate nociceptive transmission, contribute to the development of peripheral and central sensitization and possibly also contribute to pain processing and vestibular system modulation (20). Therefore, the primary mechanism of action for BTX-A in VM might be reversing peripheral and central sensitization by inhibiting the release of CGRP and (to a lesser degree) other pain-producing molecules from TVS neurons (31). In addition, it was suggested that BTX-A-mediated blockage of excitatory synapses might indirectly enhance inhibitory neurotransmission through an as yet unknown mechanism. Recently, it was reported that such a mechanism might be connected with the opioid and GABA-ergic systems in the central nervous system (32). Therefore, we speculate that the primary mechanism of BTX-A in patients with VM might involve the inhibition of TVS neuropeptide release such as CGRP and the enhancement of endogenous opioidergic and GABA-ergic transmissions, which can modulate neural activity within both the TVS and the vestibular system. Supportively, BTX-A has been shown to be less effective in chronic tension-type headache, a condition in which CGRP levels have been shown to be normal (33).

Cortical areas involved in processing vestibular information include the insular and retroinsular cortex, the parietal operculum, the STG, the temporo-parietal junction and posterior parietal cortex, the postcentral gyrus, the cingulum, the hippocampus, and the frontal cortices (34). These regions are multisensory areas in which the inputs of several sensory modalities, i.e., auditory, somatosensory, and visual, converge with vestibular information. Similarly, pain is a complex, multifactorial subjective experience, and a large distributed brain network is involved in nociceptive processing (35). Nociceptive-specific cortical and subcortical brain regions that are commonly activated by pain stimulation include the anterior cingulate cortex, insula cortex, frontal and pre-frontal cortices, primary and secondary somatosensory cortices, thalamus, basal ganglia, cerebellum, amygdala, hippocampus, and regions within the parietal and temporal cortices (36). The somatosensory and nociceptive cortical projections strongly overlap with the vestibular cortical projections, giving rise to vestibular-somatosensory and vestibular-nociceptive interactions, whose occurrence has been demonstrated by caloric and galvanic vestibular stimulation in humans (37). Therefore, it has been suggested that vestibular input could influence the processing of other tactile and nociception inputs.

In this study, alterations in spontaneous brain activity in VM patients were evaluated before and after BTX-A treatment using two different sequential data-driven analyses, namely, ALFF and a seed-based functional connectivity (FC) analysis. Increased spontaneous activity was indicated by significantly higher ALFF values in the right temporal lobe (STG) after BTX-A treatment compared with the baseline. FC between the right STG and left PSG, right SMG, and right MTG was also increased after treatment. The enhanced regional functional activity after BTX-A treatment, mainly in the right temporal lobe, which contains the vestibular cortical core regions and participating pain processing regions, suggests that BTX-A injection can modulate the functional connectivity of the vestibular and pain cortices. The STG is an important area within the vestibular network and is closely connected to other multisensory brain regions. The posterior STG is consistently described as a core region for central vestibular processing, along with the posterior insula and parietal operculum, and as a secondary processing region for coordination of the eyes and head and spatial orientation. Structural and functional abnormalities of the STG have previously been demonstrated in both migraine patients (38) and patients with vestibular disorders (39). In chronic migraine, hypometabolism was reported in the bilateral temporal regions compared with healthy controls (38). On the contrary, hypermetabolism in the temporal lobe regions, including the temporal pole and STG, has been found in response to painful stimuli (40). In VM patients, an increased metabolism in the temporal areas was reported during a migraine attack (41). During the attack-free interval of VM patients, voxel-based morphometry in MRI showed a decrease in GM volume bilaterally in the inferior and middle temporal gyri, the cingulate cortex, and the posterior insula, as well as in the STG, supramarginal gyrus, dorsolateral prefrontal cortex, and inferior occipital gyrus (39). In this study, alterations in the ALFF values were observed only at the right side STG after treatment, which reflects the right-hemispheric dominance of the vestibular cortical network in right-handers in our VM patients (42).

The FC between the right STG and the right SMG increased after BTX-A treatment, and interestingly, the correlation analysis showed a negative correlation with the headache-related disability in the MIDAS score. The SMG and angular gyrus within the inferior parietal lobule (IPL) have been shown to be activated by vestibular stimulation (43). The IPL is also part of somatosensory processing, functions in the pain response and the sensing of temperature and pressure. In patients with migraine with or without aura, structural abnormalities of the SMG have been demonstrated (44). VM patients also have decreased GM volume in the SMG, the prefrontal cortex, and the posterior insula–operculum regions compared with healthy controls. Increased FC between the right STG and SMG and the negative correlation with the MIDAS score indicate that migrainous headaches improved after BTX-A treatment over time in parallel with the increased FC between the STG and SMG. We also found increased FC between the right STG and the PCG and MTG. These brain regions belong to the multisensory vestibular network, and the areas are activated by exposure to magnetic vestibular stimulation (45). The MTG in particular is considered to be functionally relevant to vestibular compensatory mechanisms and is strongly interconnected with other multisensory cortical areas that form a multisensory-integrative network (39). The increase in FC between the STG and regions of the MTG and PCG thus allows for two interpretations: first, it contributes to vestibular compensation during the recovery phases after BTX-A treatment, and second, the inclusion of somatosensory processing might reflect the integration between the vestibular and nociceptive systems.

This study has several limitations. First, the number of VM patients is relatively small in this longitudinal approach. Second, we choose a controlled before-after study which is a non-randomized design in that outcomes are measured before and after BTX-A treatment, both in a group that receives the treatment and in another comparison group. Typically this design could have a high risk of bias because of other differences between the groups that are being compared (confounders), and the placebo effect cannot be excluded. Third, it is difficult to assert that the change in the resting-state brain connectivity is a VM-specific finding because the comparative analysis was not conducted with a group of patients with migraine other than VM. Fourth, even though we did not change the use of preventive medications, we cannot exclude the possibility that the use of preventive therapies might have influenced the resting state of the brain and led to functional changes. Lastly, the follow-up of the patients for 2 months after the treatment might be too short; longer longitudinal studies are needed to evaluate the long-term effects of BTX-A in patients with VM.

In conclusion, our novel findings that BTX-A is effective in improving vertigo and migraine attacks in patients with VM and improved functional brain activity and connectivity in the vestibular and somatosensory (pain) processing regions after BTX-A treatment are intriguing and promising to expand our understanding of the pathophysiology of VM. With respect to the therapeutic mechanisms, botulinum toxin might enhance intrinsic vestibular, nociceptive, and cross-modal interactions within the multisensory vestibular cortical network in patients with VM. The findings suggest a disease-related dysfunction of multisensory (nociceptive, somatosensory, vestibular) cortical networks in VM patients.

## Data availability statement

The original contributions presented in the study are included in the article/supplementary material, further inquiries can be directed to the corresponding authors.

## Ethics statement

This study was approved by the Ethics Committee of Jeonbuk National University Hospital (IRB File No. 2020-10-033-001). The patients/participants provided their written informed consent to participate in this study.

## Author contributions

S-YO was involved in the study concept and design and drafting a significant portion of the manuscript. J-JK was involved in the acquisition and analysis of data and drafting a significant portion of the manuscript and figures. SK and J-ML were involved in the acquisition and analysis of data. J-SK and MD were involved in the analysis of data and drafting a significant portion of the manuscript.

## Funding

This study was supported by the Neurological Disorder Research Program of the National Research Foundation of Korea (NRF) grant funded by the Korean government (Ministry of Science and ICT) (Nos. 2022R1A2B5B01001933 and 2020M3E5D9080788) and by the Fund of the Biomedical Research Institute, Jeonbuk National University Hospital.

## Conflict of interest

The authors declare that the research was conducted in the absence of any commercial or financial relationships that could be construed as a potential conflict of interest.

## Publisher's note

All claims expressed in this article are solely those of the authors and do not necessarily represent those of their affiliated organizations, or those of the publisher, the editors and the reviewers. Any product that may be evaluated in this article, or claim that may be made by its manufacturer, is not guaranteed or endorsed by the publisher.
